# Carbon quantum dots from silymarin extraction for multi-fluorescent sensors, photocatalysis, and anticancer activity applications

**DOI:** 10.1038/s41598-025-20441-8

**Published:** 2025-09-30

**Authors:** Salah Elkun, M. Ghali, T. Sharshar, M. M. Mosaad

**Affiliations:** 1 Physics Department, Faculty of Science, Kafrelsheikh University, Kafr El-Sheikh, 33516 Egypt; 2 Institute of Basic and Applied Sciences, Egypt-Japan University of Science and Technology, New Borg Al-Arab, Alexandria 21934 Egypt

**Keywords:** SM-CQDs, Silymarin, Fluorescent sensors, Photocatalysis, Anticancer activity, Quantum dots, Photonic devices

## Abstract

In this work, water-soluble fluorescent carbon quantum dots were prepared by a one-stage hydrothermal method via a green synthesis route using silymarin extract from milk thistle seeds as a single precursor. Various experimental techniques were used to characterize the synthesized silymarin-based carbon quantum dots (SM-CQDs), to confirm their structure and investigate their potential applications in fluorescent sensors, photocatalysis and anticancer activity. The prepared SM-CQDs exhibited amorphous graphitic structure with a spherical shape and an average particle size of 4.6 ± 0.7 nm, as indicated by XRD, Raman, and TEM measurements. The FTIR results indicate the presence of diverse functionalities on the surface of the SM-CQDs, which were further confirmed by XPS analysis. Fluorescence characterization of the prepared SM-CQDs revealed blue emission with a fluorescence quantum yield (QY) value of 4.9%. Furthermore, the prepared dots exhibit temperature-sensitive photoluminescence (PL) behavior, which has been interestingly used to design economical, green, and highly sensitive fluorescent probes for the detection of Fe^3+^ ions and H_2_O_2_ with a detection limit (DL) of 0.071 and 0.159 µM, respectively. A nano thermosensor has been demonstrated to have a wide temperature range of 10–90 °C and a good recovery, exhibiting a thermal sensitivity of 0.8% °C^− 1^ based on its temperature-sensitive behavior. Moreover, we demonstrated that the as-prepared SM-CQDs can serve as excellent photocatalysts for the degradation of methylene blue (MB) dye under direct visible light irradiation with a degradation efficiency of about 45% within 105 min. The obtained SM-CQDs have a zeta potential of -22.24 mV, indicating excellent stability in water. Finally the SM-CQDs exhibit anticancer activity showing cytotoxicity to Caco2 cells with IC_50_ = 285.2 ± 11.9 µg/mL. These features of SM-CQDs indicate their potential in applications such as sensing, cell imaging, and optoelectronics.

## Introduction

Carbon quantum dots (CQDs) are a new type of zero-dimensional carbon nanoparticles, consisting of quasi-spherical and discrete nanoparticles with dimensions less than 10 nm in diameter^1^. Typically, intrinsic CQDs consist of the elements carbon and oxygen. Its structure consists of sp^2^ and sp^3^ hybridized carbon atoms and organic functional groups on the surface of the carbon atoms^2^. CQDs exhibit unique chemical and physical properties such as high water solubility, nano-size effect, easy surface functionalization, excellent biocompatibility, high fluorescence quantum yield, controllable photoluminescence (PL) behavior, wide Stokes shift, and low toxicity^3^. In previous research studies with these properties, CQDs have been presented as alternatives to heavy metal-based quantum dots (QDs), organic dyes, and other carbon-based nanomaterials. They are now prominent in various applications, such as biomedical sensors (fluorescent probes), light-emitting diodes (LED), and photocatalytic devices due to their optical properties^4^. However, due to their limited fluorescence quantum yields and few active sites for modification, many efforts have been made to enhance their luminescent properties. Doping with heteroatoms has been demonstrated to be a practical way for improving the fluorescence quantum yields of CQDs.

Nowadays, many methods have been developed to synthesize fluorescent carbon quantum dots, such as hydrothermal/solvothermal treatment^5^, arc discharge^6^, laser ablation^7,8^, electrochemical^5^, ultrasonic^9^, and microwave-assisted^10^. Among these methods, hydrothermal processing is one of the most efficient and low-cost green chemistry methods for synthesizing a wide range of fluorescent CQDs from biomass resources as carbon sources^11^. In addition, this method is easy to prepare and produces uniformly shaped particles with high yield^12^. In this work, we synthesize fluorescent CQDs using silymarin extraction from milk thistle seeds. Milk thistle plant (scientific name: *Silybum marianum L. Gaertn*) is an important medicinal plant. Therefore, this plant is an annual plant native to the Mediterranean. It grows wild throughout Europe, North Africa, the Americas, and Australia, but can also be cultivated^13^. The plant’s seeds contain a group of flavonoids commonly known as silymarin. Content and composition of the main silymarin components (silybin, isosilybin, silydianin and silychristin), these groups are considered a rich source of carbon and oxygen^14^.

To our knowledge, the hydrothermal approach has not been previously used to synthesize CQDs from milk thistle (silymarin) seeds. Moreover, silymarin-based carbon quantum dots (SM-CQDs) have been applied in various applications such as fluorescent sensors for Fe^3+^ ions, H_2_O_2_, and temperature as well as in photocatalysis and as an anticancer agent. The importance of fluorescent sensors is well known in various fields, including biology, industry, and environment. In addition to everyday applications such as food and beverage analysis and the medical field, fluorescent probes are essential for scientific research. Heavy metals are pollutants that pose risks to humans, animals, and plants. One of the most important heavy metals is iron, which exists primarily in two oxidation states: Fe^2+^ and Fe^3+^. Many ecosystems and biological systems depend on iron. However, excess iron can lead to various disorders in many internal organs, including the heart, lungs, and pancreas^15,16^. Therefore, the detection of Fe^3+^ is of crucial importance. Several conventional techniques are available for the detection of metal ions, including atomic absorption spectroscopy^17^, potentiometric sensors^18^, electrochemical methods^19^, and atomic fluorescence spectroscopy^20^. However, these techniques have limitations such as high costs, long processing times, and the need for sophisticated instruments. As a result, there has been a shift toward fluorescent-based sensors, which are more cost-effective, faster, and easier to use. Several fluorescent-based sensors for detecting metal ions have been documented in the literature^21–30^.

Hydrogen peroxide (H_2_O_2_) plays a vital role as an oxidizing, bleaching, and sterilizing agent in both the biochemical and chemical industries^31^. However, high concentrations of H_2_O_2_ can irritate dyes and skin, posing risks to human health^32^. Conversely, accurately determining the concentration of H_2_O_2_ is crucial for various applications in food safety, clinical settings, and environmental monitoring^33^. Several quantitative methods are available for analysis of H_2_O_2_ in solution, including electrochemical^34^ and spectroscopic techniques^35^. While electrochemical methods are particularly effective for measuring lower concentrations of H_2_O_2_, they can be affected by interference from other reactive oxygen species. Furthermore, electrochemical detection methods are vulnerable to environmental factors, especially significant electromagnetic interference. In contrast, spectroscopic detection methods can be classified into two main types: chemiluminescence^36^ and photoluminescence^37^. These spectroscopic techniques are effective for detecting hydrogen peroxide (H_2_O_2_) at low concentrations. Therefore, our CQDs enable the development of a highly sensitive fluorescent sensor. The use of luminescent SM-CQDs offers several advantages, including small size, high selectivity, and resistance to electromagnetic interference.

Dyes are commonly used in various industries, including food, textiles, pharmaceuticals, and cosmetics, to enhance the color of products and make them more attractive to customers. However, depending on their chemical composition and use, some dyes can pose toxic risks to aquatic organisms. Industrial wastewater primarily from cosmetics, textiles, paper, and food industries can significantly pollute water sources. These pollutants can pose life-threatening risks to aquatic life, plants, and humans, even at low concentrations, due to harmful byproducts generated during their degradation, particularly through photolysis. In the textile industry, some azo dyes have been identified as degrading into toxic aromatic amines, which can lead to cancer and other health issues. Dyes cannot be eliminated from effluents using conventional processes due to their low biodegradability and complex structures^38^. Consequently, it is essential to find more efficient and cost-effective methods. Among several techniques currently used for the removal of dyes from effluents, the following can be highlighted: Fenton reaction^39^, gels and hydrogels^40^, modified membranes^41^, ultrafiltration^42^, and photocatalytic degradation^43^. Photocatalytic degradation is a widely explored effluent treatment method due to its low cost, simple operation, and high efficiency^44^. Our novel SM-CQDs can be used as photocatalysts for the degradation of organic drugs and organic dyes as methylene blue (MB)^45^.

Furthermore, colorectal carcinoma is the most common type of primary colon cancer and is known as the third fatal cancer, after lung and breast cancer^46^. A few decades ago, colorectal cancer was relatively uncommon and not frequently diagnosed^47^. Surprisingly, it is now the fourth most dangerous cancer worldwide, causing to nearly 900,000 deaths each year^48^. Besides dietary habits in developed and developing countries, obesity, lack of physical exercise, smoking, and sedentary lifestyle are major causative factors for colorectal cancer^49^. Advances in our understanding of pathophysiology have expanded the range of treatment options available. These options include local endoscopic resection and surgery, preoperative radiotherapy and systemic therapy, extended surgery for local and metastatic disease, local ablative therapies for metastases, palliative chemotherapy, targeted therapy, immunotherapy, etc^33^. Although these new therapeutic approaches have doubled the overall survival rate, survival is limited to non-metastasized diseases^50,51^. Carbon-based nanomaterials are among the most dynamic fields and are considered highly suitable for research and practical biomedical applications^52,53^.

Here, we report the preparation of CQDs from silymarin as the sole carbon source, and we systematically examine the fluorescence of SM-CQDs for their potential in sensing, photocatalysis and anticancer activity. The anticancer property of the SM-CQD was evaluated in the colorectal cancer cell line (Caco-2).

## Experimental details

### Materials

Silymarin was purchased from Sigma Aldrich, quinine hemisulfate salt monohydrate (C_20_H_24_N_2_O_2_·0.5H_2_O_4_S·H_2_O), sulfuric acid (H_2_SO_4_), methylene blue dye, C_16_H_18_N_3_SCl, (MB). Caco-2 cell lines (obtained from American Type Culture Collection (ATCC, Rockville, MD)), MTT and trypan blue dye were purchased from Sigma (St. Louis, Mo., USA). Fetal bovine serum, RPMI-1640, HEPES buffer solution, L-glutamine, gentamycin and 0.25% Trypsin-EDTA were purchased from Lonza (Belgium). Various metal ions were introduced by adding soluble metal salts (NaCl, KCl, FeSO_4_, CuSO_4_, CoSO_4_, ZnSO_4_, NiSO_4_, CdSO_4_, BaSO_4,_ CaSO_4_, Fe(NO_3_)_3_, Zn(NO_3_)_3_, and K_2_CrO_4_). All chemicals were used as received and deionized water was used throughout this study.

### Preparation and characterization of SM-CQDs

**Fig. 1:**
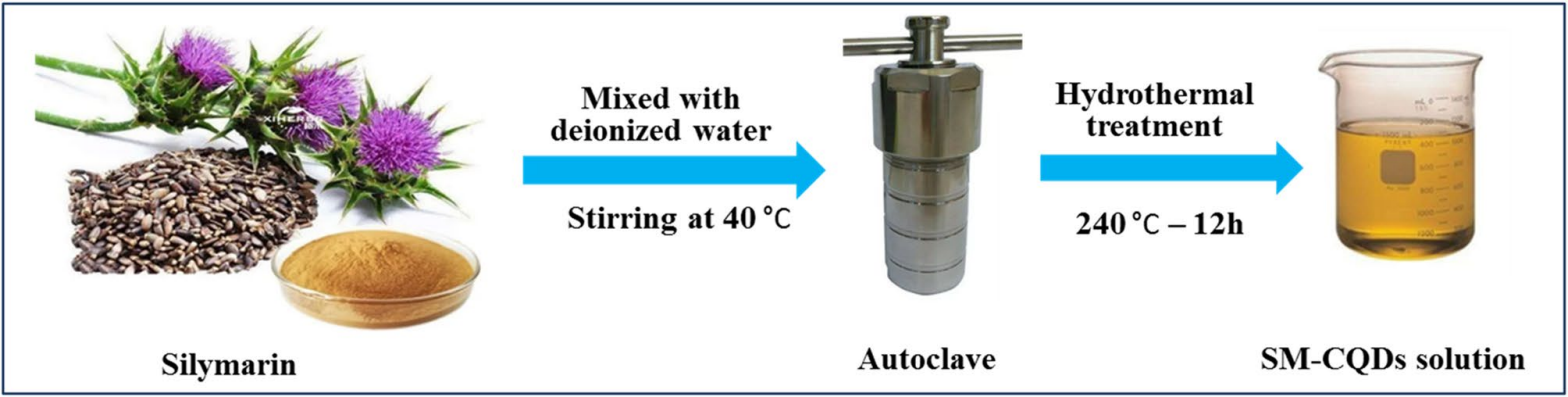
Scheme of the synthesis of silymarin-based carbon quantum dot (SM-CQDs) from milk thistle seeds by a simple hydrothermal method.

Fluorescent silymarin-based carbon quantum dots (SM-CQDs) were prepared from silymarin through a simple hydrothermal treatment process, as shown in Fig. 1. Initially, 0.15 g of silymarin powder was mixed homogeneously with 50 mL of deionized water. The solution was stirred for 2 h at 40 °C, then transferred to a Teflon-lined stainless-steel autoclave and heated at 240 °C for 12 h. After the reaction was completed, the yellowish-brown transparent product was naturally cooled at room temperature. Large insoluble particles were filtered using a 0.22 μm syringe filter membrane and dialyzed using a dialysis bag (1 kDa) for 24 h under continuous magnetic stirring against 250 mL deionized water to remove reaction residues and byproducts. The resulting solution of deionized water with dispersed SM-CQDs was stored at a temperature of below 4 °C. Finally, a portion of the SM-CQDs solution was freeze-dried under vacuum to obtain a solid brown powder. This powdered product has been used for further characterization and applications.

An X-ray diffractometer (XRD-6100, Shimadzu, Japan) with Cu-Kα radiation (λ = 0.15418 nm) was used to characterize the XRD pattern of the as-prepared SM-CQDs. The synthesized SM-CQDs were characterized using Fourier transform infrared spectroscopy (FTIR-6800, JASCO, Japan) in the range of 500 to 4000 cm-1, Raman spectroscopy (WITec’s Raman microscopealpha300 R, Germany) with excitation wavelength (785 nm, Max. 133 mW), and transmission electron microscopy (TEM, JEM-2100 F, JEOL). UV/Vis absorption spectrum of SM-CQDs was measured by a UV-Vis spectrophotometer (2600, Shimadzu, Japan) in the wavelength range of 200 to 1400 nm, and photoluminescence was examined by a spectrofluorometer (Jasco FP-8300 FL, Japan) in the wavelength range of 200 to 750 nm.

The surface analysis of SM-CQDs was investigated by X-ray photoelectron spectroscopy (XPS) using a photoelectron spectrometer (Themo Fisher Scientific, USA) with Al Kα at 1350 eV. The surface charge of SM-CQDs was estimated by Zeta potential using the phase analysis light scattering (PALS) method (NanoBrook Zeta Potential Analyzer, USA) with a scattering angle of 15º and Zeta potential range (-500 to 500 mV). The charge carrier concentration of SM-CQDs film was estimated by Hall effect using an Ecopia system with a magnetic field about 0.53 T (model: HMS-3000, Korea). The cytotoxicity of the prepared SM-CQDs against Caco-2 cell lines was studied using the standard MTT assay as mentioned in “MTT assay”.

A photocatalytic chamber (50 × 45 × 50 cm^3^) with an aluminum-reflective inner surface was used for the photodegradation of methylene blue dye (MB). The applied source of photons (ELIOS LED bulb lamb, 45 W = 5000 lm output) was located at the top of the chamber. The distance between the light source and the surface of the dye solution was ~ 5 cm. The beaker, containing the 100 cm^3^ of MB dye solution 10 µM (3.2 ppm) and 2 mL (12 ppm) of the as-prepared SM-CQDs catalyst, was put inside the dark photocatalytic chamber. The measurement was carried out at room temperature and a solution pH value of 7. The SM-CQDs were continuously mixed in dye solution using magnetic stirring. The light was turned on after 30 min to achieve the adsorption equilibrium. The initial dye concentration was determined before solution illumination by measuring the initial absorbance intensity (*A*_0_). Then, the illuminated solution’s dye concentration was determined by measuring the final absorbance intensity (*A*_*t*_) at 15, 30, 45, … and 105 min, after light turned on. The absorbance intensity was measured at the MB dye maximum absorption (*λ* = 664 nm) using the previously mentioned UV–Vis spectrophotometer. The photo-degradation efficiency of MB dye was calculated using the equation, $\:\text{P}\text{h}\text{o}\text{t}\text{o}\text{d}\text{e}\text{g}\text{r}\text{a}\text{d}\text{a}\text{t}\text{i}\text{o}\text{n}\:\left(\text{\%}\right)=\left[{(A}_{0}-{A}_{t})/\:{A}_{0}\right]\times\:100)\:$^54^.

### MTT assay

The cytotoxic concentration (IC_50_), the concentration required to inhibit 50% of intact cells, was examined for SM-CQDs on the human colorectal cancer (Caco-2) cell line (HTB-37) using MTT assay. Caco-2 cell lines were cultured in RPMI-1640 medium (modified, with sodium bicarbonate, without L-glutamine and phenol red, liquid, sterile-filtered, suitable for cell culture) supplemented with 10% phosphate-buffered saline (FBS) and 1% antibiotic (50 µg/ml gentamycin). The cultures were maintained in a humidified atmosphere at 37 °C in 5% CO_2_ and 95% relative humidity in a biochemical incubator. The culture media were replaced at least twice a week. Caco-2 was seeded in 96-well microplates to a final concentration of (1 × 105) cells/well containing 100 µL of cell line-specific medium and incubated at 37 °C in a 5% CO_2_ atmosphere. The cells were then treated with SM-CQDs at concentrations ranging from (3.9–1000 µg/mL) of SM-CQDs and incubated at 37 °C for 24 h. Subsequently, the cells were subjected to MTT analysis to determine cell viability^55^. Optical density was measured at 590 nm on a microplate reader (Sunrise, TECAN, Inc, USA) with control wells containing only cell culture medium. The IC_50_ was estimated using dose-response curve histograms for each concentration using GraphPad Prism software (San Diego, CA., USA). On the other hand, cell morphology was observed using an inverted microscope (CKX41; Olympus, Japan) equipped with a digital microscopy camera to capture the images representing morphological changes compared to control cells.

## Results and discussion

### Structural and composition of SM-CQDs

#### XRD, Raman and FTIR measurements

**Fig. 2:**
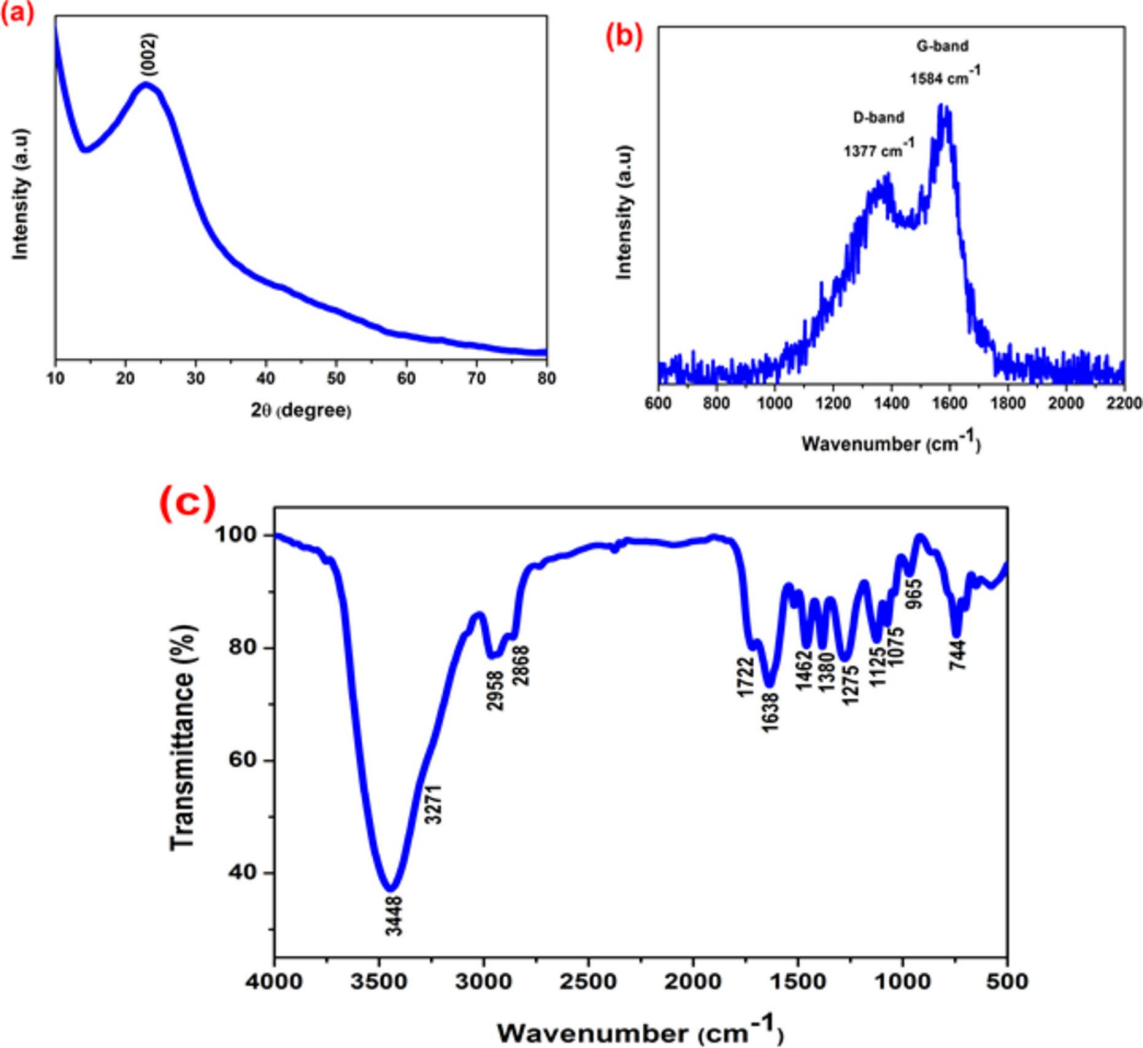
(**a**) XRD pattern, (**b**) Raman spectra, and (**c**) FTIR spectrum of the synthesized SM-CQDs.

The typical XRD pattern of the as-prepared SM-CQDs is shown in Fig. 2(a), with an intense broad peak centered at *2θ* = ∼23°. This peak is attributed to the (002) plane of the graphitic structure of carbon dots. Furthermore, the XRD results also confirmed that SM-CQDs have a predominantly graphitic structure with an interlayer spacing *(d)* of 0.36 nm, which is larger than that of the graphite to the (002) plane^56^. The increase in interlayer spacing results from the steric hindrance of surface functional groups at the edge of graphite, plane distortion due to (sp^3^) carbon in the graphite structure, and defects introduced by various heteroatoms^57^. Therefore, the XRD results indicate the presence of SM-CQDs with amorphous nature^58,59^.

Meanwhile, Raman spectra have been performed to explore the structure of SM-CQDs. As shown in Fig. 2(b), the Raman spectrum of SM-CQDs exhibits two major bands at 1377 and 1584 cm^−1^, which can be attributed to D-band (sp^3^) and G-band (sp^2^), respectively. In general, the D band represents the vibration mode of the carbon atom in the sp^3^ hybridized orbital and the G band is associated with the vibration mode of sp^2^ carbon atom. The relative intensity of the disorder D-band and the crystalline G-band (*I*_*D*_*/I*_*G*_) in this work is approximately about 0.74, which is characteristic of the disorder’s amorphous nature and the ratio of sp^3^/sp^2^ carbon. This result indicates a significant level of graphitization in SM-CQDs, corroborated by the XRD data^60^.

FTIR spectroscopy was used to identify the functional groups present on the surface of the SM-CQDs. The FTIR spectrum of the synthesized SM-CQDs is shown in Fig. 2(c). The observed broad bands at 3448 and 3271m^−1^ were attributed to O-H and N-H stretching vibrations, respectively. The bands at 2958 and 2868 m^−1^ are assigned to the asymmetric and symmetric stretching of the C-H bonds, respectively. The bonds of C = O, C = C, C-N/C-P, C-OH, and C-O-C functional groups were observed at 1722, 1638, 1275, 1125, and 1075 m^− 1^, respectively. The absorption band located at 1380 m^− 1^ was assigned to the bending vibration of the C–H bond^61,62^. Furthermore, Ca-CO_3_ and Ca-O can be further observed at 1462 and 744 cm^−1,^ respectively^63^. Finally, the peak at 965 cm^− 1^ identified the presence of a P-O phosphate bond in SM-CQDs^64^. The observation of numerous FTIR bands can be attributed to the hydrothermal method, which facilitates the dehydration and decomposition of the precursor, ultimately leading to the formation of various carbon skeletons^65^.

#### Morphological study

**Fig. 3:**
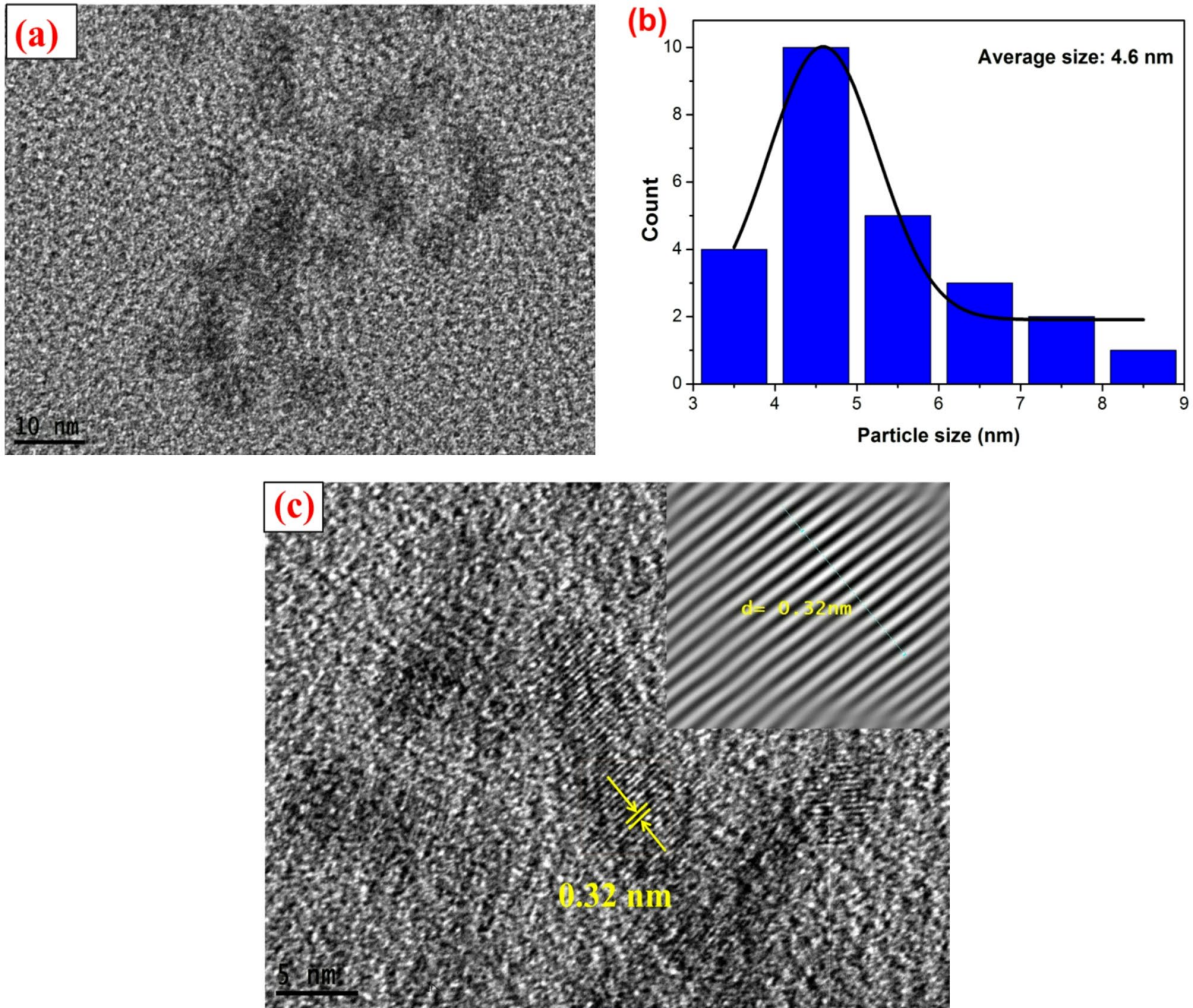
(**a**) TEM image, (**b**) histograms of particle size values with their Gaussian distribution (solid line), and (**c**) HR-TEM image of SM-CQDs.

The morphological properties of the prepared SM-CQDs are shown in the TEM image ( see Fig. 3). The TEM image reveals that the SM-CQDs have a quasi-spherical shape with uniform dispersion. Moreover, as shown in Fig. 3(b), the average particle size of SM-CQDs is 4.6 ± 0.7 nm with a size distribution range from 3.05 to 8.10 nm. The HR-TEM image, Fig. 3(c), showed that the inter-planer spacing of the SM-CQDs sample is 0.32 nm, which is consistent with the graphitic structure of carbon dots^66^.

#### XPS analysis

**Fig. 4:**
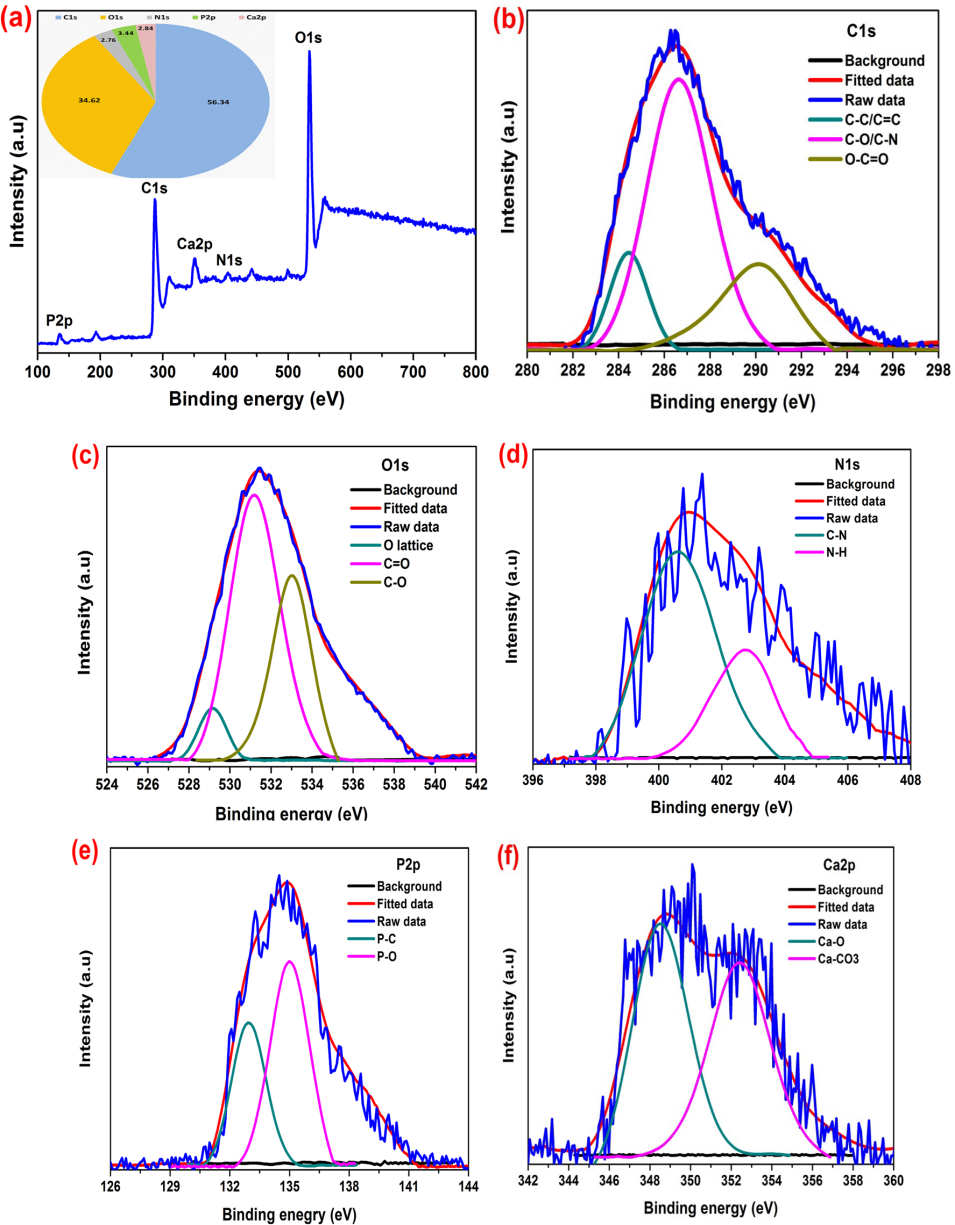
(**a**) XPS survey spectra of SM-CQDs. (**b**) High resolution XPS spectra of C1s, (**c**) O1s, (**d**) N1s, (**e**) P2p, and (**f**) Ca2p.

The XPS survey spectrum as well as the high-resolution scans of C, O, N, P and Ca on the surface of SM-CQDs are shown in Fig. 4. The XPS survey spectrum, Fig. 4(a), of the SM-CQDs sample showed five distinct peaks at 287.3, 534.3, 403.7, 135.4 and 350.8 eV, which are attributed to C1s, O1s, N1s, P2p and Ca2p, respectively. The content of C, O, N, P and Ca elements was determined from the survey spectrum to be 56.34, 34.62, 2.76, 3.44 and 2.84%, respectively. In Fig. 4(b), the C1s peak deconvoluted into three peaks at 284.4, 286.6 and 290.3 eV originating from C–C/C = C, C–O/C–N/C–P and O-C = O groups, respectively^67^. Figure 4(c) represents the O1s spectrum with peaks at 529.2, 531.1 and 533.1 eV which are assigned to the binding energies of metal oxide (O^2−^ anions of the crystalline network)^68^, C = O and C–O, respectively^69^. The N1s band of the SM-CQDs, Fig. 4(d), can be fitted into two peaks at 400.6 and 403.4 eV, which are assigned to C–N and N–H bonds, respectively. The P2p band of the SM-CQDs, Fig. 4(e), can be fitted into two peaks at 132.9 and 134.9 eV, which are assigned to P–C and P–O bonds, respectively. The Ca2p band of SM-CQDs (Fig. 4(f)) can be fitted into two peaks at 348.5 and 352.4 eV, which are assigned to Ca–O and Ca–CO_3_ bonds, respectively^70^. The XPS results are consistent with the results of the FTIR measurements, and both confirm the presence of various functionalities on the surface of the SM-CQDs. Surface configurations include functionalities, edge states, and defects while heteroatom doping involves doping the conjugated aromatic domains with various atoms. These configurations endow the SM-CQDs with not only excellent solubility in water, but also fluorescence emission^71,72^.

### Optical properties

#### Absorption of SM-CQDs

**Fig. 5:**
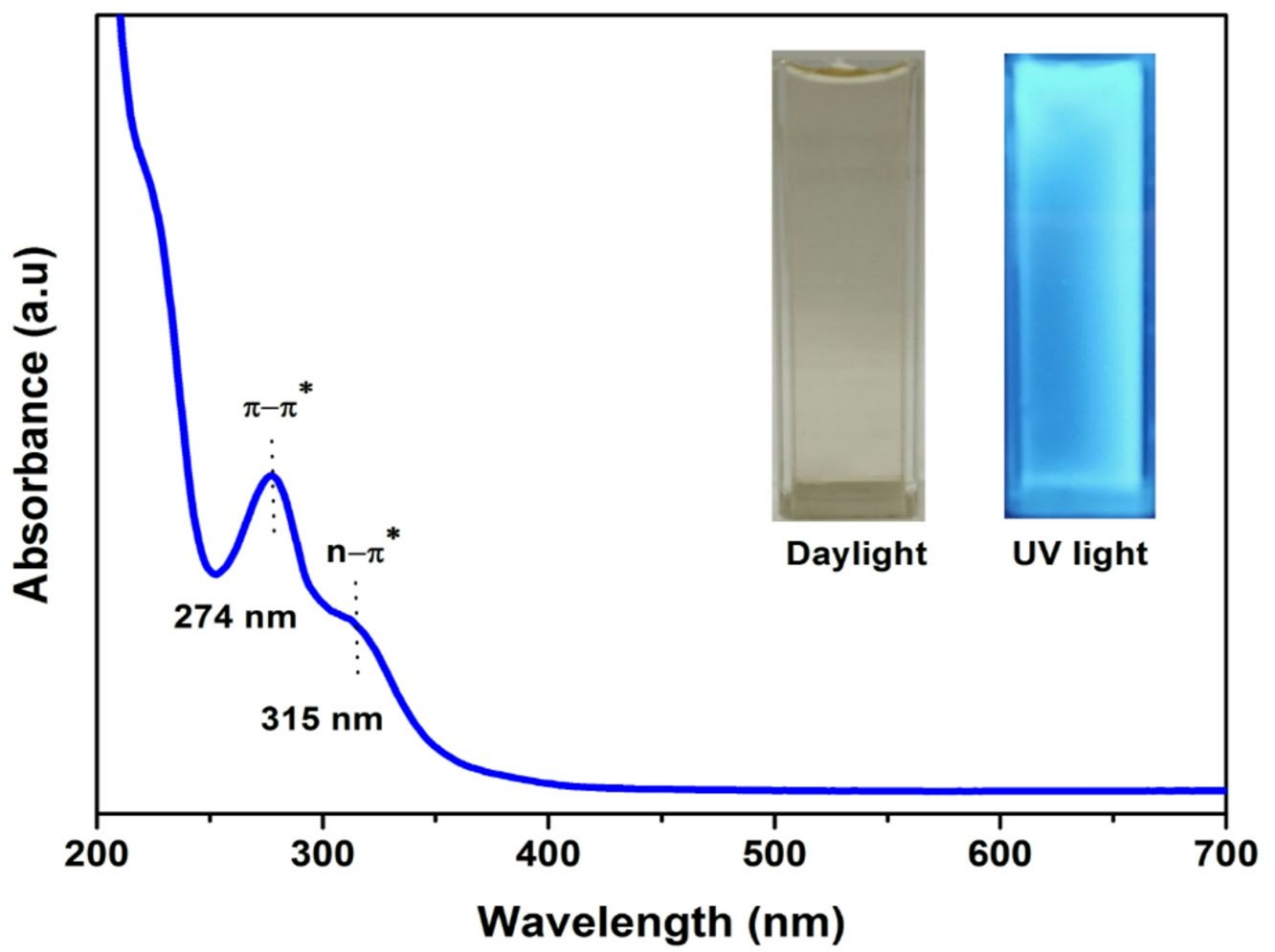
UV − Vis absorption spectrum (the insets show the photographs of SM-CQDs solution under daylight (left) and 365 nm UV light (right)).

The UV-Vis absorption spectrum of the prepared SM-CQDs is shown in Fig. 5. There are two main absorption peaks with maximum absorption at 274 and 315 nm in the UV region. The absorption peaks at 274 and 315 nm are associated with the typical π–π* and n-π* transitions of aromatic bonds (C = C) and the transition of surface functional groups as (C = O), respectively^65^. The inset of Fig. 5 shows photographs of SM-CQDs under visible daylight (left) and UV illumination (right). These results reveal that the solution showed bright blue fluorescence under UV (365 nm) irradiation, confirming the presence of carbon dots, and the pale yellow color of SM-CQDs was observed with daylight irradiation.

#### Fluorescence of SM-CQDs solution

**Fig. 6:**
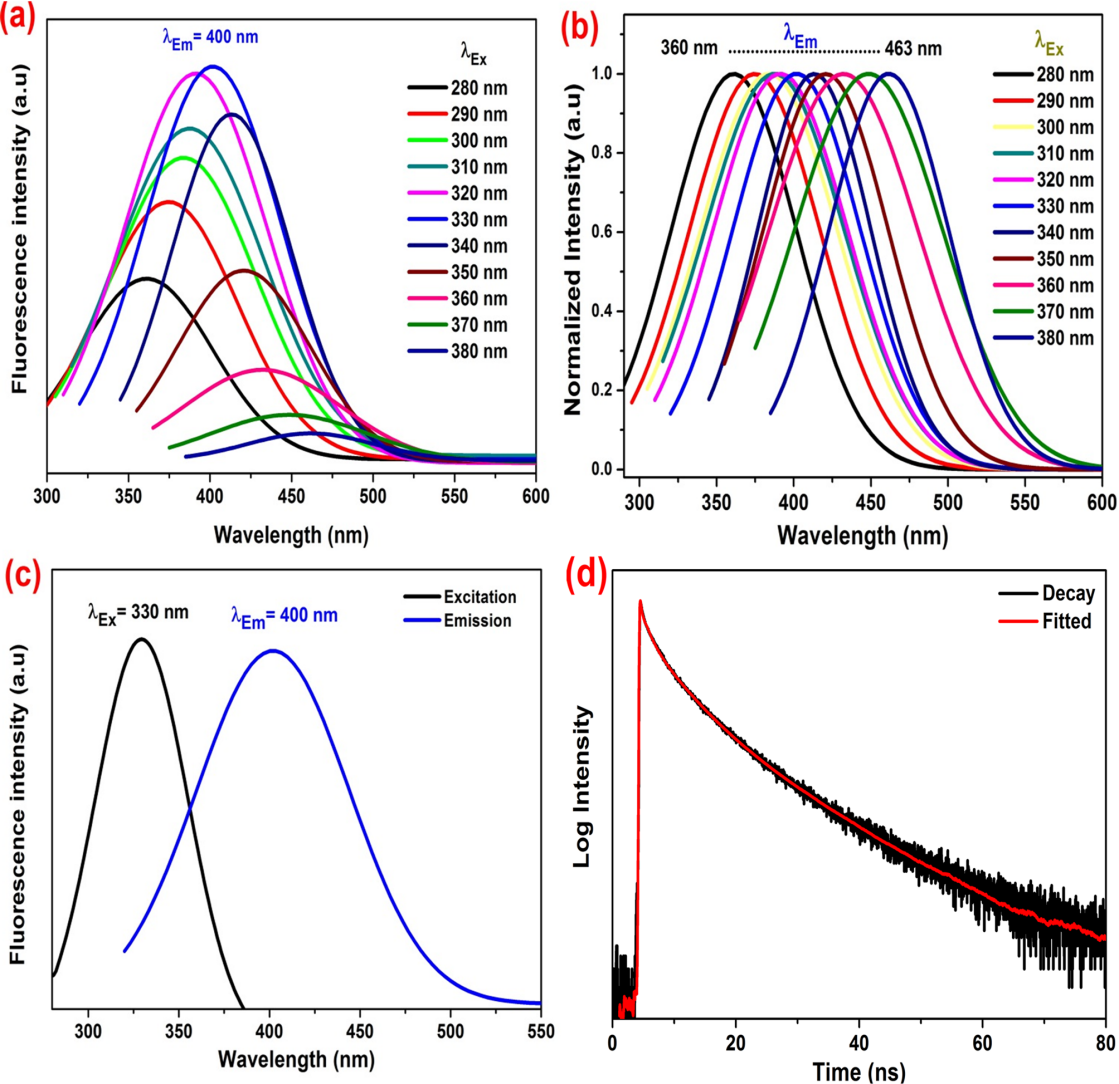
(**a**) PL spectra of prepared CQDs under different excitation wavelengths, (**b**) normalized PL spectra at different excitation wavelengths, (**c**) excitation and emission spectra, and (**d**) fluorescence decay curves of SM-CQDs.

Figure 6 illustrates the photoluminescence spectra (PL) of the prepared SM-CQDs sample. In Fig. 6(a), the PL spectra are presented under different excitation wavelengths ranging from 280 to 380 nm. As the excitation wavelength increases, the fluorescence emission peak shifts from 360 nm to 463 nm, as depicted in Fig. 6(b) of the normalized PL spectra. Notably, when the excitation wavelength increases from 280 to 330 nm, the fluorescence intensity gradually increases, reaching a maximum at 330 nm, before it declines as the excitation wavelength increases from 330 to 380 nm. The maximum fluorescence emission intensity was at λ_Em_ = 400 nm when the SM-CQDs were excited by light of λ_Ex_ = 330 nm, as shown in Fig. 6(c). These results indicate a typical excitation-dependent emission behavior of silymarin-based carbon quantum dots^73^. The fluorescence of SM-CQDs was found to decay in a monoexponential manner, resulting in an average fluorescence lifetime of 6.4 ns (see Fig. 6(d)). To our knowledge, this relatively long lifetime is rarely observed in carbon dots derived from natural sources. Therefore, the prepared SM-CQDs are suitable for sensing and optoelectronics applications^74^.

The fluorescence quantum yield (QY) of the synthesized SM-CQDs was calculated according to the following relationship^75^:

$$\:Q{Y}_{CQDs}=Q{Y}_{R}\left[\frac{\frac{dI}{d{A}_{CQDs}}}{\frac{dI}{d{A}_{R}}}\right]\left(\frac{{n}_{CQDs}^{2}}{{n}_{R}^{2}}\right)$$

where *I* is the integrated fluorescence intensity under the fluorescence emission spectrum, QY_CQDs_ *and* QY_*R*_ represent the fluorescence quantum yield of the test substance and reference compound. Quinine sulfate dissolved in 0.1 M H_2_SO_4_ (QY_*R*_ = 0.54) was chosen as a reference. *n* is the refractive index (1.33 for aqueous solution). *A* is the absorbance at the excitation wavelength of 330 nm. The quantum yield was found to be 4.9% at an excitation wavelength of 330 nm.

### Electrical properties of SM-CQDs

#### Zeta potential analysis

**Fig. 7:**
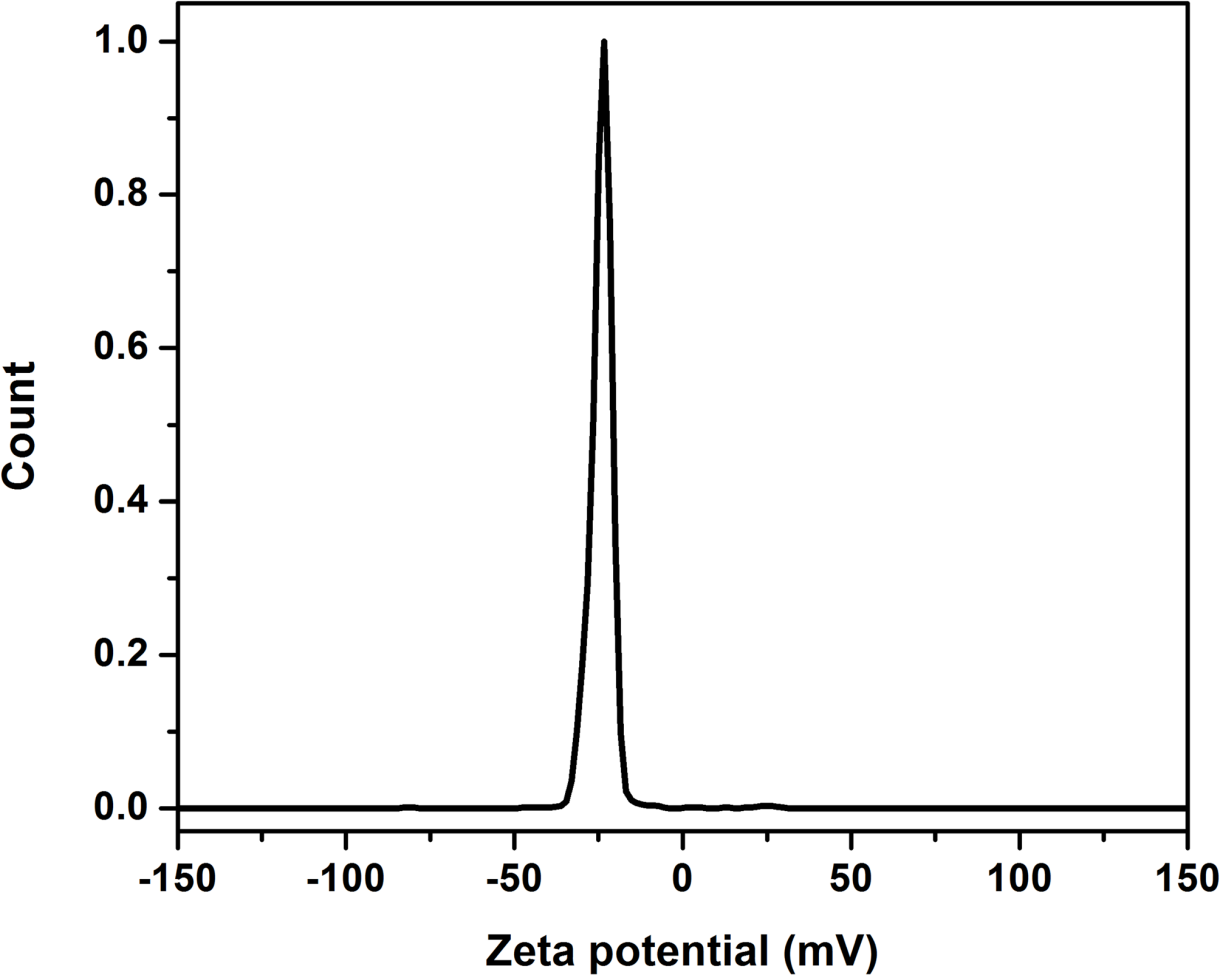
Zeta Potential spectrum of the prepared SM-CQDs.

Zeta potential is a key indicator for assessing the stability of carbon quantum dots in water by measuring the electrical charge surrounding their surface. The zeta-potential spectrum of the prepared CQDs is shown in Fig. 7. A high zeta potential value (-22.24 mV) was found, indicating that the prepared SM-CQDs have excellent stability and good dispersibility in their colloidal solutions. The negative zeta potential values indicate that the surface of the SM-CQDs carries negative charge groups. These charged groups are crucial to indicating excellent SM-CQDs stability in a water-based solvent^76^.

#### Hall-effect measurement

The electrical properties of the ≈ 100 nm-thick SM-CQDs film were studied by room-temperature Hall effect measurements using an Ecopia system with a magnetic field about 0.53 T. The results are summarized in Table 1 and show that the SM-CQDs film has a p-type character with high carrier conductivity. The latter demonstrates the possibility of using SM-CQDs film as an efficient hole transport layer^77^.

**Table 1:** Hall-effect measurement of SM-CQDs film.

	Mobility µ [cm^2^/(V.s)]	Resistivity ρ [Ω.cm]	Carrier Concentration [1/ cm^3^]	Hall Coefficient [cm^3^/C]	Conductivity σ [1/( Ω.cm)]
CQDs film	5.94 × 10^2^	2.84 × 10^− 5^	3.70 × 10^20^	1.69 × 10^2^	3.52 × 10^4^

### Fluorescent sensors based on SM-CQDs

#### SM-CQDs as a nanothermosensor

**Fig. 8:**
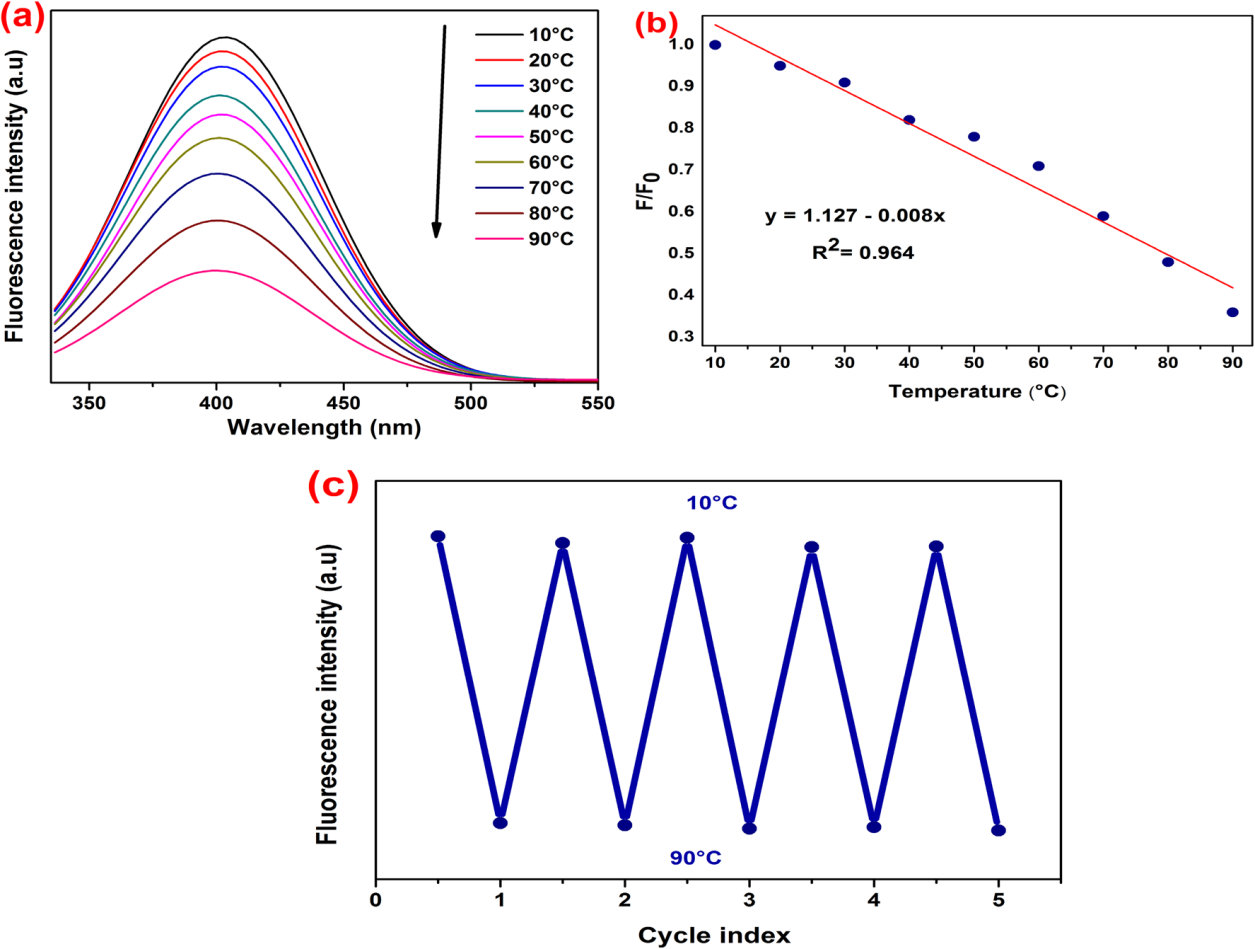
(**a**) Fluorescence spectra of SM-CQDs at different temperatures under the optimal excitation wavelength (330 nm), (**b**) linear relationship of fluorescence intensity with temperature, and (**c**) fluorescence intensity in five different cycles from 10 to 90 °C.

Figure 8 displays the fluorescence spectra of SM-CQDs at different temperatures; it was observed that the fluorescence intensity decreased significantly with temperature in the range of 10–90 °C under the optimum excitation wavelength 330 nm, as shown in Fig. 8(a). Figure 8(b) shows the normalized fluorescence intensity of SM-CQDs versus different temperatures where a linear relationship is obtained with the variance R^2^ = 0.964. No discernible shift in the emission spectra was found in the studied range. From 10 to 90 °C, the fluorescence intensity reduced by 63.9% with a thermal sensitivity of 0.8% °C^–1^. Moreover, as shown in Fig. 8(c), the current sensor exhibits exceptional reversibility and restorability when the temperature is cycled five times from 10 to 90 °C. Therefore, SM-CQDs can be an ideal temperature nanosensor with high accuracy and reproducibility^78^.

#### Fe^3+^ sensor

**Fig. 9:**
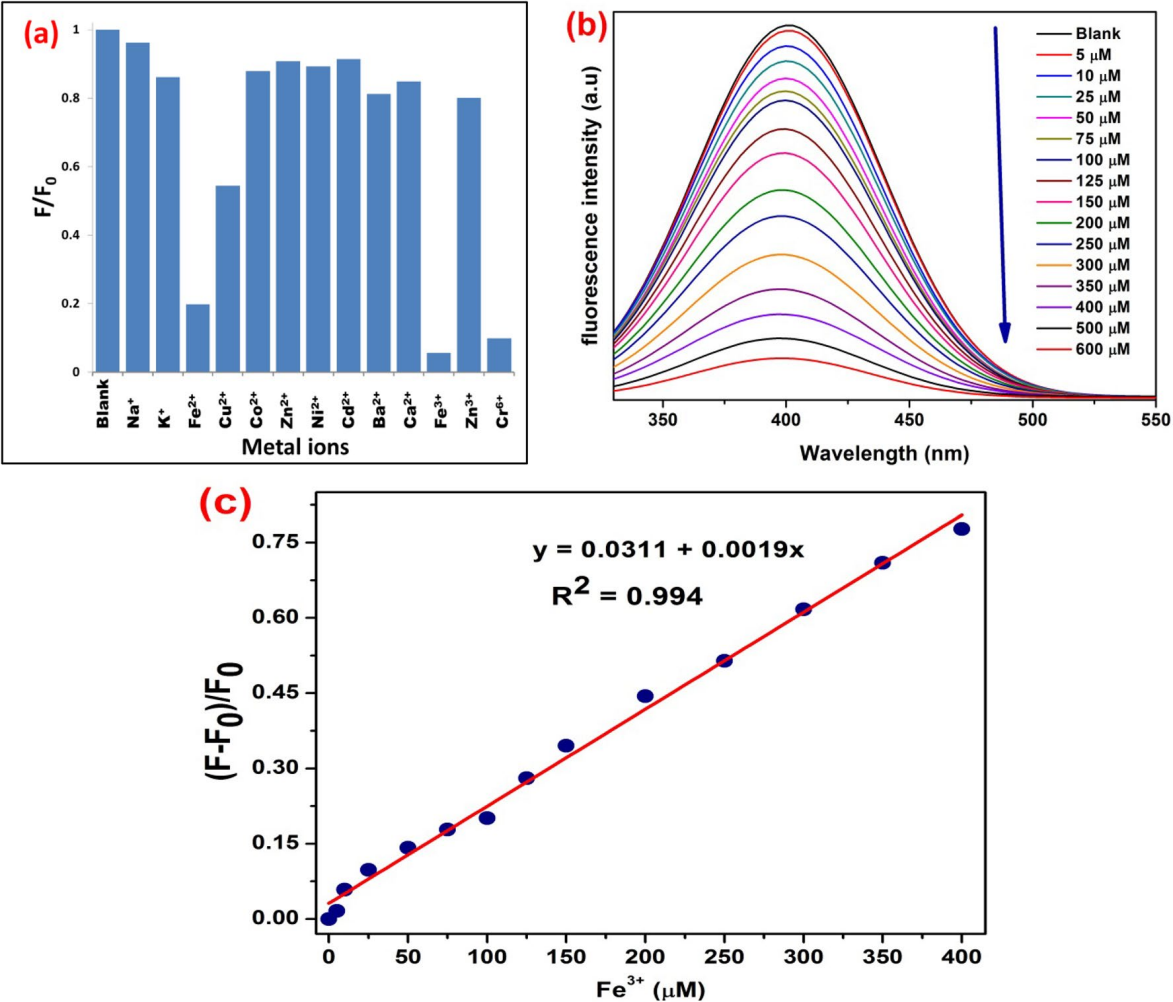
(**a**) Fluorescence intensity ratios (F/F0) of the SM-CQDs at different concentrations of metal ions, (**b**) fluorescence emission spectra of SM-CQDs in the presence of increasing Fe^3+^ ion concentrations (0–600 µM), and (**c**) the Stern–Volmer quenching profile with Fe^3+^ ion concentration ranging between 0 and 400 µM.

Fluorescence sensing of SM-CQDs was conducted using an optimal excitation wavelength of 330 nm while maintaining a constant concentration of different metal ions. The effects on the fluorescence intensity of SM-CQDs were observed after the addition of metal ions at room temperature. Figure 9(a) shows the different quenching effects caused by the metal ions Na^+^, K^+^, Fe^2+^, Cu^2+^, Co^2+^, Zn^2+^, Ni^2+^, Cd^2+^, Ba^2+^, Ca^2+^, Fe^3+^, Zn^3+^and Cr^6+^. Most of the metal ions showed little change in fluorescence intensity, while Fe^3+^ and Cr^6+^ ions caused significant fluorescence quenching of SM-CQDs. The results showed that Fe^3+^ ions had a greater effect on the fluorescence quenching of SM-CQDs than Cr^6+^; thus, the SM-CQDs sensor is selective for Fe^3+^. As shown in Fig. 9(b), under the optimized conditions, the sensitivity of SM-CQDs to Fe^3+^ was investigated, where the fluorescence spectra of SM-CQDs were recorded in the presence of Fe^3+^ ions concentrations of 0-600 µM. The Stern–Volmer quenching profile of SM-CQDs with Fe^3+^ ion concentration has a linear relationship, R^2^ = 0.994, in the Fe^3+^ concentration range of 0-400 µM (see Fig. 9(c)) and with a detection limit of 0.071 µM. The presence of Fe^3+^ can affect the surface states of quantum dots, and functional groups on SM-CQDs can be easily coordinated with Fe^3+^, resulting in higher selectivity toward Fe^3+^ due to the transfer of photoelectrons from SM-CQDs to Fe^3+^ ions^78^. It is worth noting that our SM-CQDs have a broader Fe^3+^ range compared to recently published assays based on CQDs from other precursors (see Table 2). Therefore, SM-CQDs can potentially be used as a sensor for Fe^3+^ ion detection. In addition, the detection limit value and linear range of our proposed SM-CQDs method were compared to other reported CQD-based sensors (Table 2), and as seen, our SM-CQDs provide the lowest detection limit value and wide linear range.

**Table 2:** Comparison of the sensing performance of different fluorescent probes for the detection of Fe^3+^ ions.

Fluorescent Probe	Linear Range/(µM)	DL/(µM)	Ref.
CDs from banana peels	2–16	0.21	^21^
N-CQDs	0–200	0.15	^22^
C-CQDs	2–50	1.30	^23^
CMCQDs	0.1–20	0.6	^24^
CQDs from gelatin	0–50	0.2	^25^
Rose-heart radish	0.02–40	0.13	^26^
Sweet potato	1–100	0.32	^27^
Blueberry	12–100	9.97	^28^
CQDs from Pomelo Peel	0.1–160	0.086	^29^
NCDs from Prosopis juliflora plant stems	20–200	0.593	^30^
SM-CQDs	0–400	0.071	The present work

**Fig. 10:**
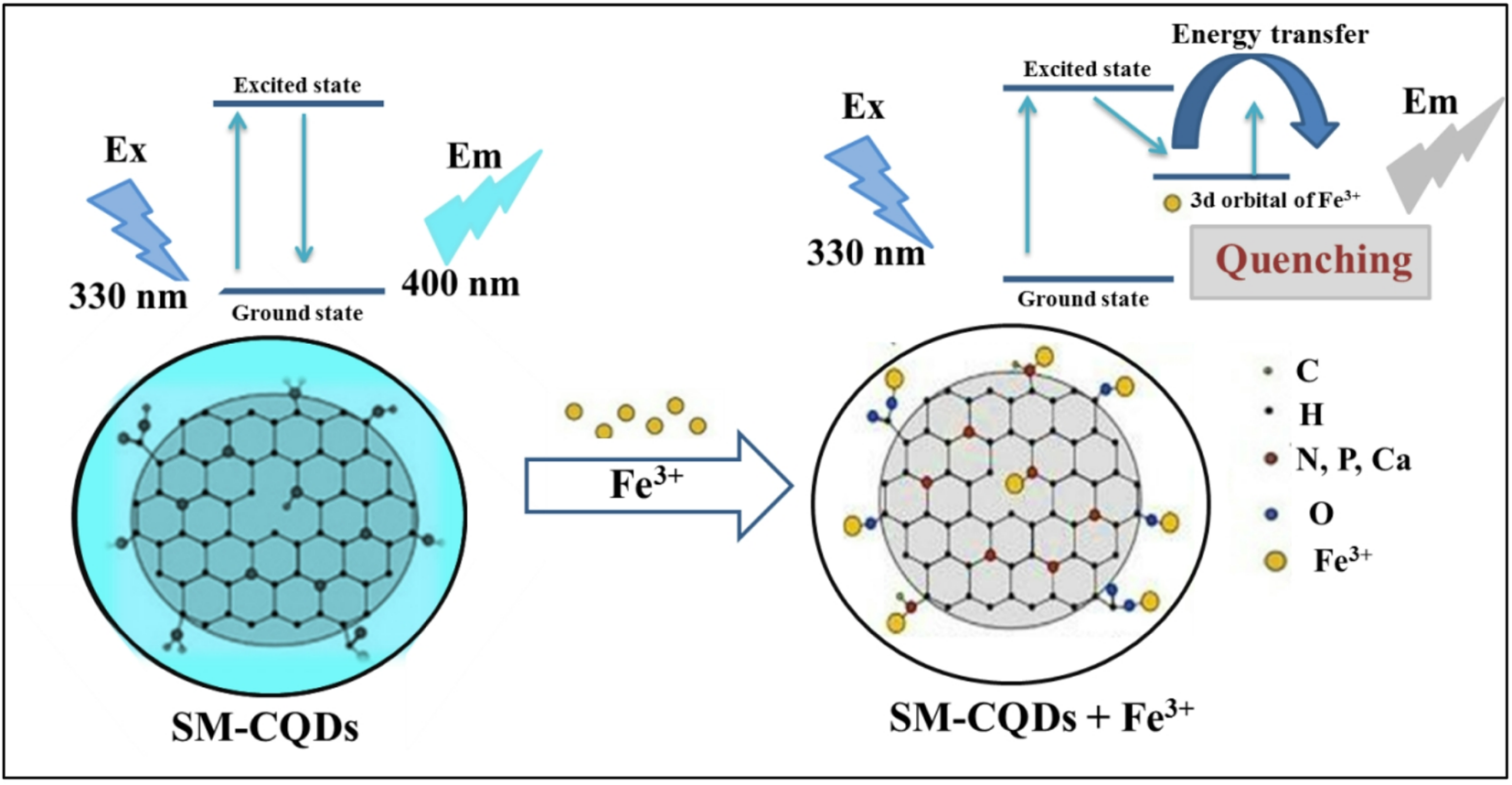
Fluorescence quenching mechanism of the SM-CQDs in the presence of Fe^3+^ ions.

It is worth noting that the strong quenching effect of SM-CQD by Fe^3+^ ions may arise from the formation of SM-CQD + Fe^3+^ complexes. A plausible mechanism for the fluorescence quenching of SM-CQD is the intramolecular photoinduced electron transfer process from excited SM-CQD to Fe^3+^ ions (see Fig. 10)^79^. Upon addition of Fe^3+^, the electron-deficient Fe^3+^ complexes with functional groups on the SM-CQD surface (e.g., carboxyl groups and amino groups) lead to the splitting of the d-orbital of Fe^3+^. Therefore, the electrons in the excited state of SM-CQDs were partially transferred to the d-orbital of Fe^3+^. Thus, electron transition was restricted to radiation forms (PL emission), resulting in fluorescence quenching^80,81^. Therefore, the above results strongly demonstrate that the SM-CQD sensor has strong selectivity for Fe^3+^ sensing^82^.

#### H_2_O_2_ sensor

**Fig. 11:**
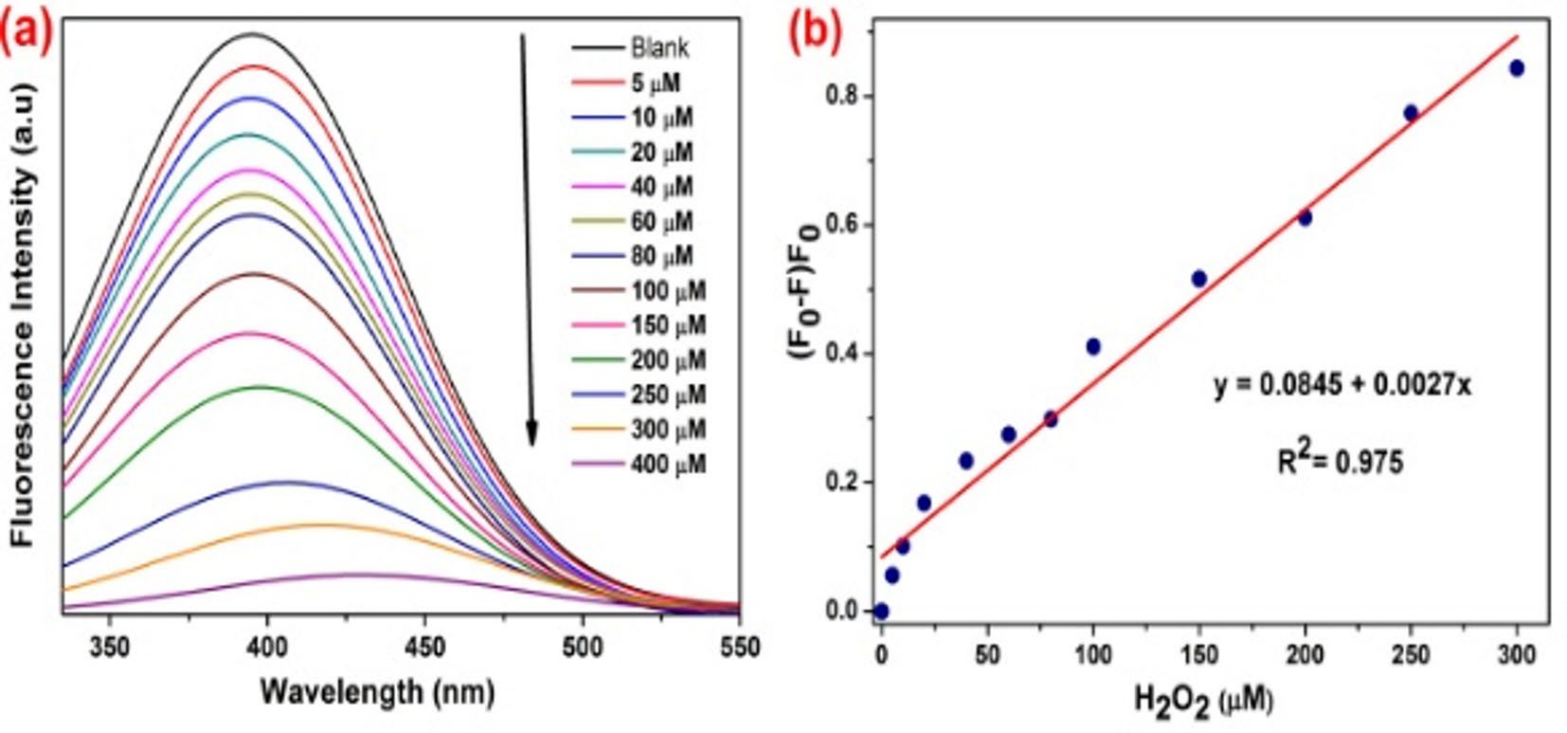
(**a**) Fluorescence emission spectra of SM-CQDs in the presence of increasing concentrations of H_2_O_2_ (0-400 µM), and (**b**) Stern–Volmer quenching profile with H_2_O_2_ concentration ranging from 0 to 300 µM.

The fluorescence responses of the synthesized SM-CQDs were measured in the presence of H_2_O_2_ with a different concentration ranging from 0 to 400 µM and the fluorescence response for each addition was recorded under the optimal excitation wavelength of 330 nm. It is interesting to note that the intensity of fluorescence spectra gradually decreases with increasing H_2_O_2_ concentration, which is shown in Fig. 11(a). As shown in Fig. 11(b), the SM-CQDs with H_2_O_2_ concentration ranging from 0 to 300 µM exhibit Stern–Volmer quenching profile. More importantly, a good linear relationship was found between the fluorescence quenching efficiency (F_0_ – F)/F_0_ and H_2_O_2_ concentration within the range of 0-300 µM as shown Fig. 11(b). The results reveal that the fluorescence intensity of SM-CQDs is strongly dependent on H_2_O_2_ concentration, R^2^ = 0.975, with a H_2_O_2_ detection limit of 0.159 µM. The overall result indicates that the synthesized SM-CQDs are an excellent fluorescence probe for H_2_O_2_ detection^83^.

### Photocatalysis activity of SM-CQDs

**Fig. 12:**
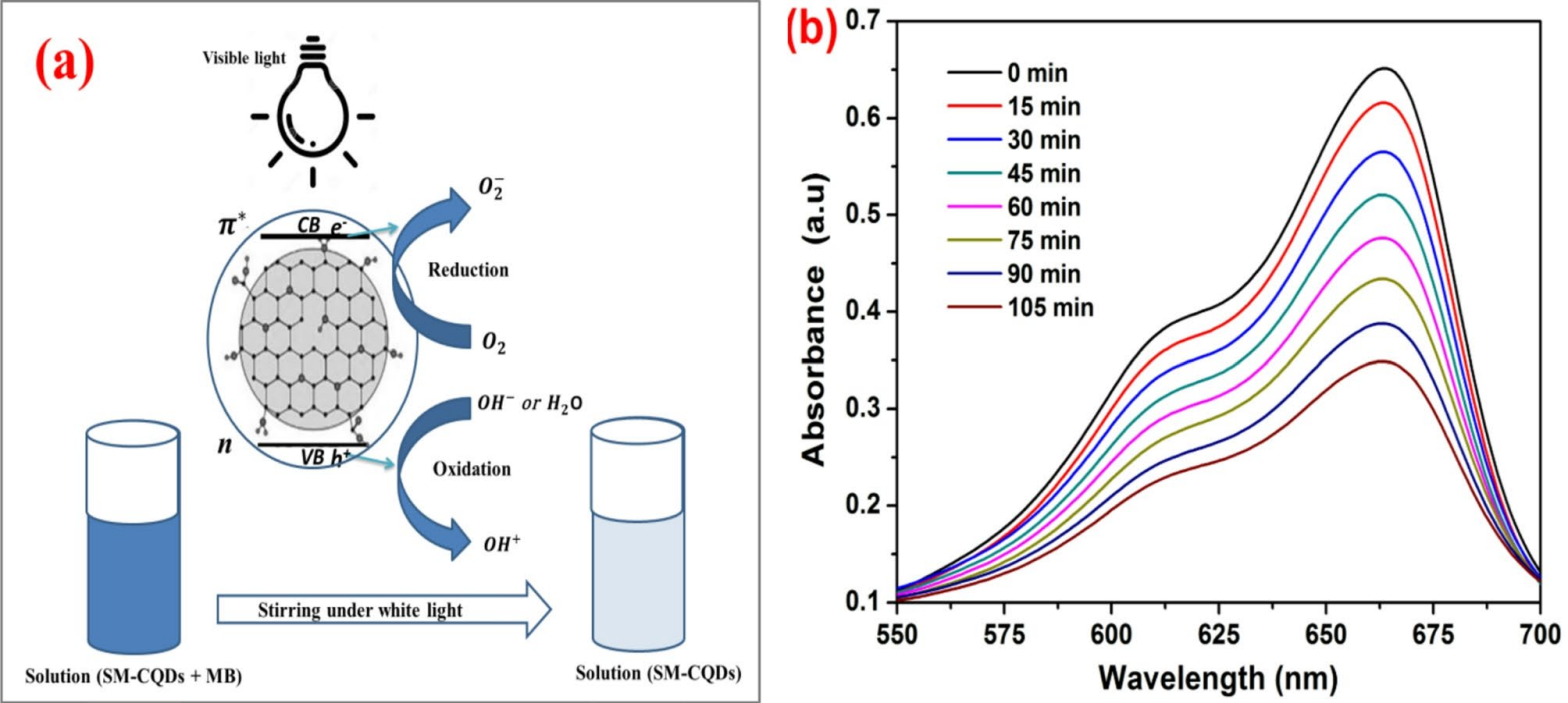
(**a**) Mechanism of the electron transfer due to photons and decolorization of MB dye and (**b**) UV–Vis spectrum for the decolorization of MB dye under white light irradiation with SM-CQDs.

A schematic representation of the photocatalytic degradation of SM-CQDs is illustrated in Fig. 12(a). By directly absorbing light, carbon dots change their chemical composition with the transfer of electrons from the HOMO to the LUMO. Carbon dots have a strong ability to absorb light in the UV–vis range due to the C = C double bond’s propensity to undergo the π-π* transition in the inner core. The outer layer of SM-CQDs contains a variety of functional groups. Most of its absorption in the visible range occurs due to the n-π* quantum confinement effect. The external light energy source excites the valence band electrons$\:{\:(e}_{CB}^{-})$, causing them to migrate to the conduction band of the SM-CQDs. As a result, this electron transfer creates a hole in the valence band^84^. The photoexcited electrons react with molecular oxygen adsorbed on the surface of the SM-CQDs, producing superoxide radical anions ($\:{O}_{2}^{-}$) and hydrogen peroxide ($\:{{H}_{2}O}_{2}^{\:}$)^85^. The photogenerated hole ($\:{h}_{\:}^{+}$) reacts with $\:{OH}^{-}$ or $\:{H}_{2}O$ oxidizing them into $\:{OH}^{*}$ radicals. OH^∗^ radicals are strong oxidizing agents that decolorize most MB dyes. Superoxide radical anions ($\:{O}_{2}^{-}$) and holes generated by electron transfer ($\:{h}_{VB}^{+}$) contribute to the decolorization of MB dye^84^.

The SM-CQDs displayed a gradual decrease in MB dye concentration over time when exposed to visible light, as shown in Fig. 12(b). The result indicated that the characteristic absorption band of MB dye centered at 664 nm gradually decreased as a function of visible light irradiation time, with a destruction efficiency of 45% within 105 min. This low photocatalytic efficiency can be significantly increased by optimizing various parameters (light type and intensity, solution pH, catalyst dose, oxidizing agent addition, etc.) affecting the photocatalytic process^86,87^.

### SM-CQDs as anticancer agent

**Fig. 13:**
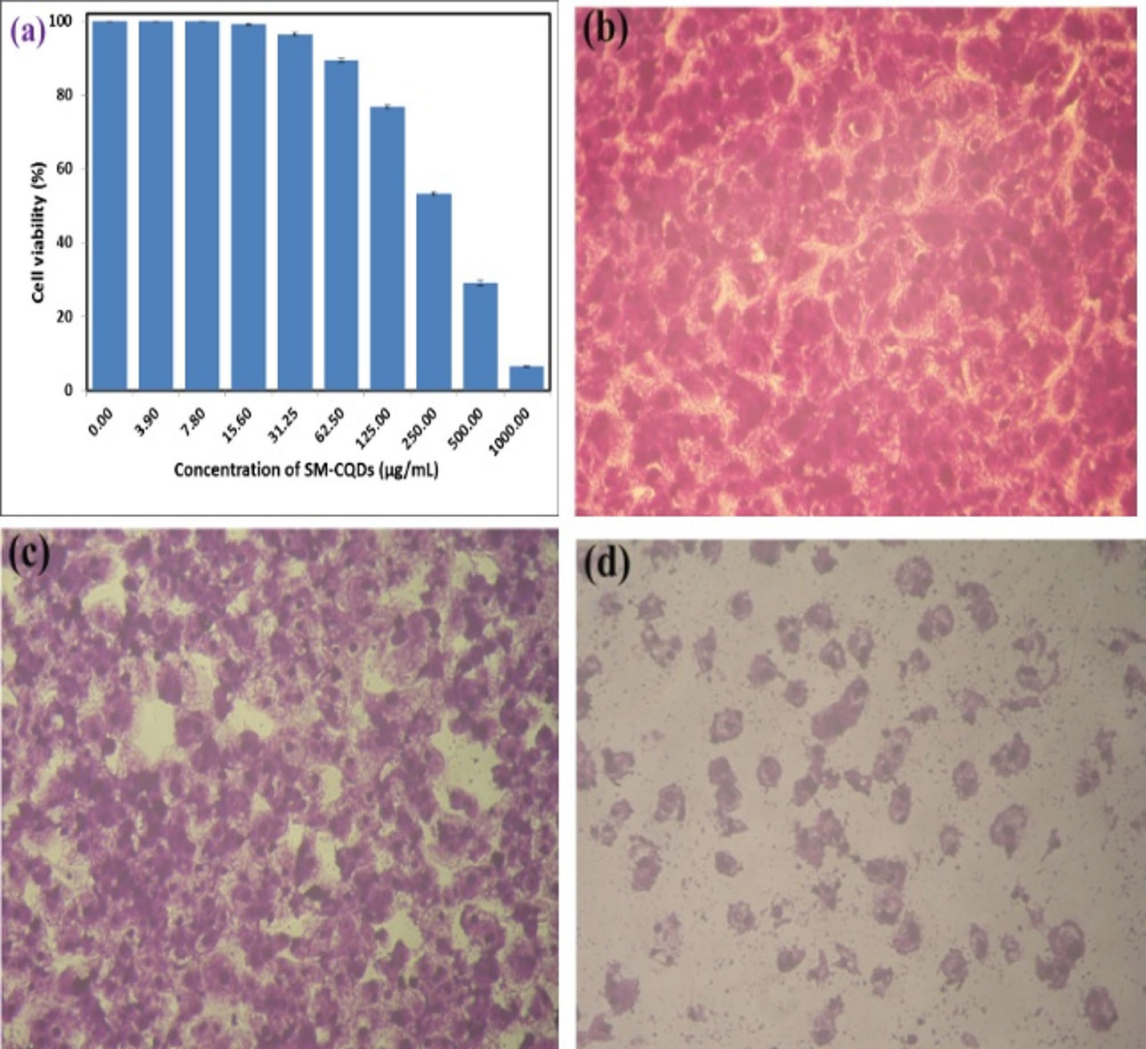
The percentage of cell viability as a function of SM-CQDs concentrations (a) and morphological images of treated Caco2 cells with the SM-CQDs concentration of zero, as a control (b), of 250 µg/mL (c) and of 1000 µg/mL (d). Images were taken at (100 μm) scale bar and 200x magnification.

MTT assay was used to perform viability testing at different concentrations of SM-CQDs ranging from 3.9 to 1000 µg/mL. A graph showing the percentage of cell viability in the cancer cell line (Caco2) as a function of the SM-CQDs concentrations, as well as morphological images of Caco2 cells treated with different concentrations of SM-CQDs is shown in Fig. 13. Figure 13(a) shows no change in cell viability until it reaches the cytotoxic inhibitory concentration (IC_50_ = 285.2 ± 11.9 µg/mL). There is a significant loss of cancer cell viability in human colorectal, even at higher concentrations of SM-CQDs. This result confirms that the synthesized SM-CQDs show good biocompatibility and are suitable candidates as anticancer agent as well as for other biomedical applications. In addition, cytopathic effects (morphological alterations) were observed microscopically at 200x, as shown in Fig. 13 (b, c, and d ; images captured by inverted microscope reveal cell inhibition after treatment with SM-CQDs.

## Conclusion

Small-size (4.6 nm) water-soluble fluorescent carbon quantum dots (SM-CQDs) were synthesized using silymarin as the carbon source via a simple hydrothermal reaction. XRD study reveals the amorphous nature of SM-CQDs in good agreement with Raman and TEM results. Several functional groups, on the surface of SM-CQDs were confirmed by FTIR spectra as well as surface analysis via XPS spectroscopy. The results showed remarkable optical properties of the prepared SM-CQDs as they can emit blue fluorescence under UV light with a quantum yield of about (4.9%). It was found that Fe^3+^ ions and H_2_O_2_ could quench the fluorescence of SM-CQDs with high sensitivity and selectivity. Remarkably, SM-CQDs also exhibited interesting temperature-dependent fluorescence behavior and proved to effectively function as a nanothermometer for monitoring intracellular temperature. Moreover, SM-CQDs exhibit moderate cytotoxicity with IC_50_ = 285.2 ± 11.9 µg/mL. It also shows photocatalysis activity with a degradation efficiency of about 45% after 105 min. The SM-CQDs are well-dispersed in colloidal form with a zeta potential of -22.24 mV. Therefore, the prepared SM-CQDs are suitable candidates for many applications, such as multi-fluorescent sensors, photocatalysis, and anti-cancer agents.

## Data Availability

All data generated or analyzed during this study are included in this published article.
